# Supplementary material for: Association between Vitamin D receptor (VDR) gene polymorphisms and hypertensive disorders of pregnancy: a systematic review and meta-analysis
Source: PeerJ. 2023 Apr 25;11:e15181. doi: 10.7717/peerj.15181 (PMC10143592; doi:10.7717/peerj.15181)
Supplement: Supplemental Information 1 [file peerj-11-15181-s001.docx]

| **Section and Topic** | **Item #** | **Checklist item** | **Location where item is reported** |
| --- | --- | --- | --- |
| **TITLE** | | |  |
| Title | 1 | Identify the report as a systematic review. | Page 1, Line 1~2 |
| **ABSTRACT** | | |  |
| Abstract | 2 | See the PRISMA 2020 for Abstracts checklist. | Page 4~5 of **Supplemental Table S1.** |
| **INTRODUCTION** | | |  |
| Rationale | 3 | Describe the rationale for the review in the context of existing knowledge. | Page 5, Line 101~106 |
| Objectives | 4 | Provide an explicit statement of the objective(s) or question(s) the review addresses. | Page 5, Line 114~116 |
| **METHODS** | | |  |
| Eligibility criteria | 5 | Specify the inclusion and exclusion criteria for the review and how studies were grouped for the syntheses. | Page 6~7, Line 137~146 |
| Information sources | 6 | Specify all databases, registers, websites, organisations, reference lists and other sources searched or consulted to identify studies. Specify the date when each source was last searched or consulted. | Page 6,  Line 125~127 |
| Search strategy | 7 | Present the full search strategies for all databases, registers and websites, including any filters and limits used. | **Supplemental Table S2** |
| Selection process | 8 | Specify the methods used to decide whether a study met the inclusion criteria of the review, including how many reviewers screened each record and each report retrieved, whether they worked independently, and if applicable, details of automation tools used in the process. | Page 7,  Line 134~136 |
| Data collection process | 9 | Specify the methods used to collect data from reports, including how many reviewers collected data from each report, whether they worked independently, any processes for obtaining or confirming data from study investigators, and if applicable, details of automation tools used in the process. | Page 7~8,  Line 147~155 |
| Data items | 10a | List and define all outcomes for which data were sought. Specify whether all results that were compatible with each outcome domain in each study were sought (e.g. for all measures, time points, analyses), and if not, the methods used to decide which results to collect. | Page 7~8,  Line 147~155 |
|  | 10b | List and define all other variables for which data were sought (e.g. participant and intervention characteristics, funding sources). Describe any assumptions made about any missing or unclear information. | Page 7~8,  Line 147~155 |
| Study risk of bias assessment | 11 | Specify the methods used to assess risk of bias in the included studies, including details of the tool(s) used, how many reviewers assessed each study and whether they worked independently, and if applicable, details of automation tools used in the process. | Page 8,  Line 156~162 |
| Effect measures | 12 | Specify for each outcome the effect measure(s) (e.g. risk ratio, mean difference) used in the synthesis or presentation of results. | Page 8~9,  Line 163~180 |
| Synthesis methods | 13a | Describe the processes used to decide which studies were eligible for each synthesis (e.g. tabulating the study intervention characteristics and comparing against the planned groups for each synthesis (item #5)). | Page 7,  Line 137~146 |
|  | 13b | Describe any methods required to prepare the data for presentation or synthesis, such as handling of missing summary statistics, or data conversions. | Page 8~9,  Line 163~180 |
|  | 13c | Describe any methods used to tabulate or visually display results of individual studies and syntheses. | Page 8~9,  Line 163~180 |
|  | 13d | Describe any methods used to synthesize results and provide a rationale for the choice(s). If meta-analysis was performed, describe the model(s), method(s) to identify the presence and extent of statistical heterogeneity, and software package(s) used. | Page 8~9,  Line 163~180 |
|  | 13e | Describe any methods used to explore possible causes of heterogeneity among study results (e.g. subgroup analysis, meta-regression). | Page 8~9,  Line 163~180 |
|  | 13f | Describe any sensitivity analyses conducted to assess robustness of the synthesized results. | Page 8~9,  Line 172~176 |
| Reporting bias assessment | 14 | Describe any methods used to assess risk of bias due to missing results in a synthesis (arising from reporting biases). | Page 8~9,  Line 163~180 |
| Certainty assessment | 15 | Describe any methods used to assess certainty (or confidence) in the body of evidence for an outcome. | Page 8~9,  Line 163~180 |
| **RESULTS** | | |  |
| Study selection | 16a | Describe the results of the search and selection process, from the number of records identified in the search to the number of studies included in the review, ideally using a flow diagram. | Page 9,  Line 183~195 |
|  | 16b | Cite studies that might appear to meet the inclusion criteria, but which were excluded, and explain why they were excluded. | Page 9,  Line 183~195 |
| Study characteristics | 17 | Cite each included study and present its characteristics. | See Table 1 in manuscript |
| Risk of bias in studies | 18 | Present assessments of risk of bias for each included study. | **Supplemental Table S3** |
| Results of individual studies | 19 | For all outcomes, present, for each study: (a) summary statistics for each group (where appropriate) and (b) an effect estimate and its precision (e.g. confidence/credible interval), ideally using structured tables or plots. | See Table 2 and 3 in manuscript |
| Results of syntheses | 20a | For each synthesis, briefly summarise the characteristics and risk of bias among contributing studies. | See Table 2 in manuscript |
|  | 20b | Present results of all statistical syntheses conducted. If meta-analysis was done, present for each the summary estimate and its precision (e.g. confidence/credible interval) and measures of statistical heterogeneity. If comparing groups, describe the direction of the effect. | Page 11~12,  Line 222~244 |
|  | 20c | Present results of all investigations of possible causes of heterogeneity among study results. | Page 11~12,  Line 222~244 |
|  | 20d | Present results of all sensitivity analyses conducted to assess the robustness of the synthesized results. | Page 12,  Line 245~252 |
| Reporting biases | 21 | Present assessments of risk of bias due to missing results (arising from reporting biases) for each synthesis assessed. | Page 12,  Line 245~252 |
| Certainty of evidence | 22 | Present assessments of certainty (or confidence) in the body of evidence for each outcome assessed. | Page 11~12,  Line 222~244 |
| **DISCUSSION** | | |  |
| Discussion | 23a | Provide a general interpretation of the results in the context of other evidence. | Page 12,  Line 255~259 |
|  | 23b | Discuss any limitations of the evidence included in the review. | Page 16~17,  Line 350~361 |
|  | 23c | Discuss any limitations of the review processes used. | Page 16~17,  Line 350~361 |
|  | 23d | Discuss implications of the results for practice, policy, and future research. | Page 16,  Line 346~349 |
| **OTHER INFORMATION** | | |  |
| Registration and protocol | 24a | Provide registration information for the review, including register name and registration number, or state that the review was not registered. | Page 6,  Line 119~120 |
|  | 24b | Indicate where the review protocol can be accessed, or state that a protocol was not prepared. | Page 6,  Line 119~120 |
|  | 24c | Describe and explain any amendments to information provided at registration or in the protocol. | Page 6,  Line 119~120 |
| Support | 25 | Describe sources of financial or non-financial support for the review, and the role of the funders or sponsors in the review. | Page 18, Line 392~393 |
| Competing interests | 26 | Declare any competing interests of review authors. | Page 18, Line 390~391 |
| Availability of data, code and other materials | 27 | Report which of the following are publicly available and where they can be found: template data collection forms; data extracted from included studies; data used for all analyses; analytic code; any other materials used in the review. | Page 18,  Line 387~389 |

PRISMA 2020 Checklist for Abstracts

| **Section and Topic** | **Item #** | **Checklist item** | **Reported (Yes/No)** |
| --- | --- | --- | --- |
| **TITLE** | | |  |
| Title | 1 | Identify the report as a systematic review. | Yes. Page 1, Line 1~2 |
| **BACKGROUND** | | |  |
| Objectives | 2 | Provide an explicit statement of the main objective(s) or question(s) the review addresses. | Yes. Page 2, Line 27~29 |
| **METHODS** | | |  |
| Eligibility criteria | 3 | Specify the inclusion and exclusion criteria for the review. | Yes. Page 2, Line 34~35 |
| Information sources | 4 | Specify the information sources (e.g. databases, registers) used to identify studies and the date when each was last searched. | Yes. Page 2,  Line 32~34 |
| Risk of bias | 5 | Specify the methods used to assess risk of bias in the included studies. | Yes. Page2,  Line 35~37 |
| Synthesis of results | 6 | Specify the methods used to present and synthesise results. | Yes. Page 2,  Line 37~39 |
| **RESULTS** | | |  |
| Included studies | 7 | Give the total number of included studies and participants and summarise relevant characteristics of studies. | Yes. Page 2,  Line 40 |
| Synthesis of results | 8 | Present results for main outcomes, preferably indicating the number of included studies and participants for each. If meta-analysis was done, report the summary estimate and confidence/credible interval. If comparing groups, indicate the direction of the effect (i.e. which group is favoured). | Yes. Page 2,  Line 40~45 |
| **DISCUSSION** | | |  |
| Limitations of evidence | 9 | Provide a brief summary of the limitations of the evidence included in the review (e.g. study risk of bias, inconsistency and imprecision). | Yes. Page 3,  Line 46 |
| Interpretation | 10 | Provide a general interpretation of the results and important implications. | Yes. Page 3  Line 47 |
| **OTHER** | | |  |
| Funding | 11 | Specify the primary source of funding for the review. | No funding. |
| Registration | 12 | Provide the register name and registration number. | Yes. Page 2, Line 32. |
