# Supplementary material for: Association between Vitamin D receptor (VDR) gene polymorphisms and hypertensive disorders of pregnancy: a systematic review and meta-analysis
Source: PeerJ. 2023 Apr 25;11:e15181. doi: 10.7717/peerj.15181 (PMC10143592; doi:10.7717/peerj.15181)
Supplement: Supplemental Information 2 [file peerj-11-15181-s002.docx]

**Table S2.** Search Strategy for Each Database

| Database (Citations) | Search strategy |
| --- | --- |
| PubMed (45) | #1 ((((((((((VDR[All Fields]) OR (vitamin d receptor gene[All Fields])) OR (vitamin D receptor[All Fields])) OR (FokI[All Fields])) OR (rs2228570[All Fields])) OR (BsmI[All Fields])) OR (rs1544410[All Fields])) OR (ApaI[All Fields])) OR (rs7975232[All Fields])) OR (TaqI[All Fields])) OR (rs731236[All Fields])  #2 ((((((genetic polymorphism[All Fields]) OR (polymorphism[All Fields])) OR (polymorphic[All Fields])) OR (polymorphics[All Fields])) OR (mutation[All Fields])) OR (variant[All Fields])) OR (single nucleotide polymorphism[All Fields])  #3 ((((((hypertensive disorders of pregnancy[MeSH Terms]) OR (pregnancy-induced hypertension[MeSH Terms])) OR (pre-eclampsia[All Fields])) OR (preeclampsia[All Fields])) OR (gestational hypertension[All Fields])) OR (Pre-Eclampsia[MeSH Terms])) OR (pregnancy disorder[All Fields])  #4 #1 AND #2 AND #3 |
| Embase (67) | #1 'vitamin d receptor' OR 'vdr protein' OR 'vdr gene'/exp OR 'vdr gene' OR 'vitamin d receptor gene'/exp OR 'vitamin d receptor gene' OR 'foki gene'/exp OR 'foki gene' OR 'apai gene'/exp OR 'apai gene' OR 'bsmi gene'/exp OR 'bsmi gene' OR 'taqi gene'/exp OR 'taqi gene'  #2 'genetic polymorphism'/exp OR 'genetic polymorphism' OR 'polymorphism'/exp OR polymorphism OR 'mutation'/exp OR mutation OR 'variant'/exp OR variant OR 'snps'/exp OR 'snps'  #3 'hypertensive disorders of pregnancy' OR 'maternal hypertension'/exp OR 'maternal hypertension' OR 'preeclampsia'/exp OR 'preeclampsia' OR 'pregnancy complication'/exp OR 'pregnancy complication' OR 'pregnancy disorder'  #4 #1 AND #2 AND #3 |
| Web of Science (63) | #1 (((TS=(VDR)) OR TS=(vitamin D receptor )) OR TS=(vitamin d receptor gene)) OR TS=(vitamin d)  #2 ((TS=(genetic polymorphism)) OR TS=(polymorphism)) OR TS=(genotype)  #3 (((TS=(pregnancy-induced hypertension)) OR TS=(gestational hypertension)) OR TS=(gestational hypertensive disorders)) OR TS=(pre-eclampsia)  #4 #1 AND #2 AND #3 |
| the Cochrane Library (2) | #1 (vitamin d): ti,ab,kw OR (VDR):ti,ab,kw (Word variations have been searched)  #2 (polymorphisms): ti,ab,kw (Word variations have been searched)  #3 (preeclampsia): ti,ab,kw OR (gestational hypertension):ti,ab,kw (Word variations have been searched)  #4 #1 AND #2 AND #3 |
