# Supplementary material for: Association between Vitamin D receptor (VDR) gene polymorphisms and hypertensive disorders of pregnancy: a systematic review and meta-analysis
Source: PeerJ. 2023 Apr 25;11:e15181. doi: 10.7717/peerj.15181 (PMC10143592; doi:10.7717/peerj.15181)
Supplement: Supplemental Information 3 [file peerj-11-15181-s003.docx]

**Table S3.** Risk of bias in the included studies assessed by the Newcastle-Ottawa scale.

| Study | Selection | | | | Comparability | Exposure | | | Score | Risk of bias |
| --- | --- | --- | --- | --- | --- | --- | --- | --- | --- | --- |
|  | Is the case definition adequate | Representativeness of the cases | Selection of Controls | Definition of Controls | Comparability of cases and controls on the basis of the design or analysis | Ascertainment of exposure | Same method of ascertainment for cases and controls | Non-Response rate |  |  |
| Rezende et al., 2012 | Clear diagnostic criteria were presented (★) | Consecutive cases (★) | Hospital controls | No history of disease (★) | Study controls for the most important factors (★) | Secure record (★) | Yes (★) | Not mentioned | 6 | Medium |
| Zhan et al., 2015 | Clear diagnostic criteria were presented (★) | Consecutive cases (★) | Hospital controls | No history of disease (★) | Study controls for any additional factors (★★) | Secure record (★) | Yes (★) | Not mentioned | 7 | Low |
| Rezavand et al., 2019 | Clear diagnostic criteria were presented (★) | Consecutive cases (★) | Hospital controls | No history of disease (★) | Not in accordance with HWE | Secure record (★) | Yes (★) | Not mentioned | 5 | Medium |
| Caccamo et al., 2020 | Clear diagnostic criteria were presented (★) | Consecutive cases (★) | Hospital controls | No history of disease (★) | Study controls for any additional factors (★★) | Secure record (★) | Yes (★) | Not mentioned | 7 | Low |
| Mashhadi et al., 2020 | Clear diagnostic criteria were presented (★) | Consecutive cases (★) | Hospital controls | No history of disease (★) | Study controls for the most important factors (★) | Secure record (★) | Yes (★) | Not mentioned | 6 | Medium |
| Stola et al., 2021 | Clear diagnostic criteria were presented (★) | Consecutive cases (★) | Hospital controls | No history of disease (★) | Study controls for any additional factors (★★) | Secure record (★) | Yes (★) | Not mentioned | 7 | Low |
| Ghorbani et al., 2021 | Clear diagnostic criteria were presented (★) | Consecutive cases (★) | Hospital controls | No history of disease (★) | Study controls for the most important factors (★) | Secure record (★) | Yes (★) | Not mentioned | 6 | Medium |
| Setiarsih et al.,2022 | Clear diagnostic criteria were presented (★) | Consecutive cases (★) | Hospital controls | No history of disease (★) | Study controls for the most important factors (★) | Secure record (★) | Yes (★) | Not mentioned | 6 | Medium |
| Si et al., 2022  **(Cohort study)** | Clear diagnostic criteria were presented (★) | Consecutive cases (★) | Community controls (★) | No history of disease (★) | Study controls for the most important factors (★) | Secure record (★) | Yes (★) | Complete follow up (★) | 8 | Low |
| Aziz et al., 2022 | Clear diagnostic criteria were presented (★) | Consecutive cases (★) | Hospital controls | No history of disease (★) | Study controls for the most important factors (★) | Secure record (★) | Yes (★) | Not mentioned | 6 | Medium |
