# Supplementary figures and images for: Association between Vitamin D receptor (VDR) gene polymorphisms and hypertensive disorders of pregnancy: a systematic review and meta-analysis
Source: PeerJ. 2023 Apr 25;11:e15181. doi: 10.7717/peerj.15181 (PMC10143592; doi:10.7717/peerj.15181)

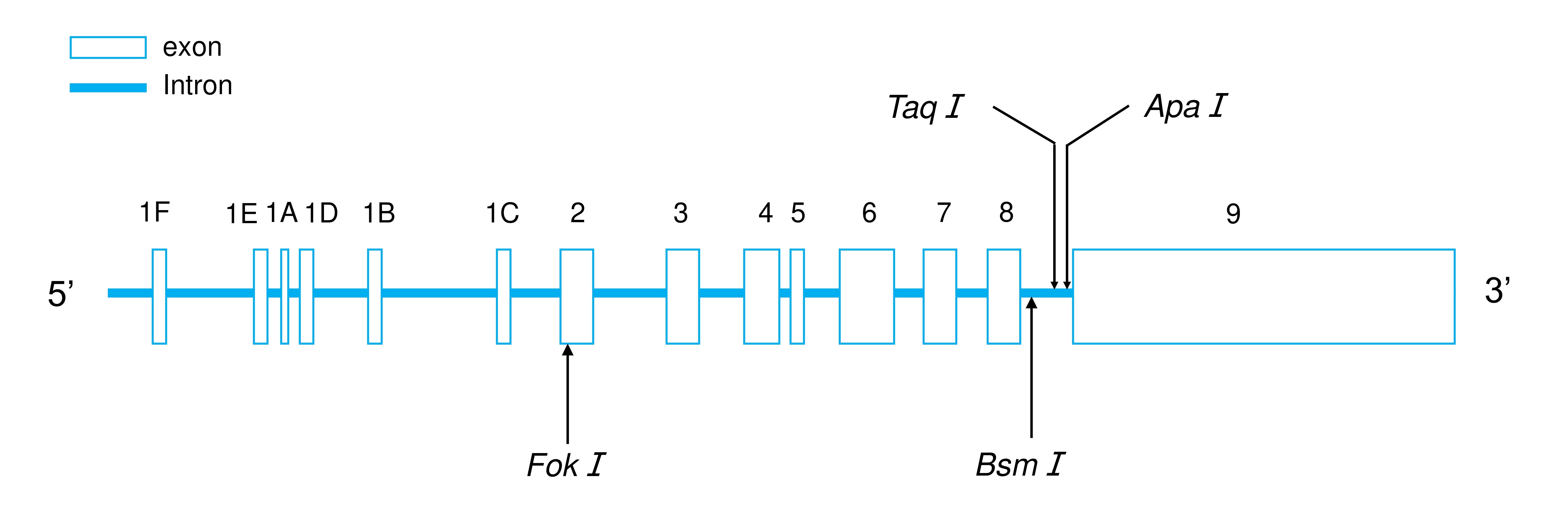

Supplement: Supplemental Information 4 [file peerj-11-15181-s004.jpg]

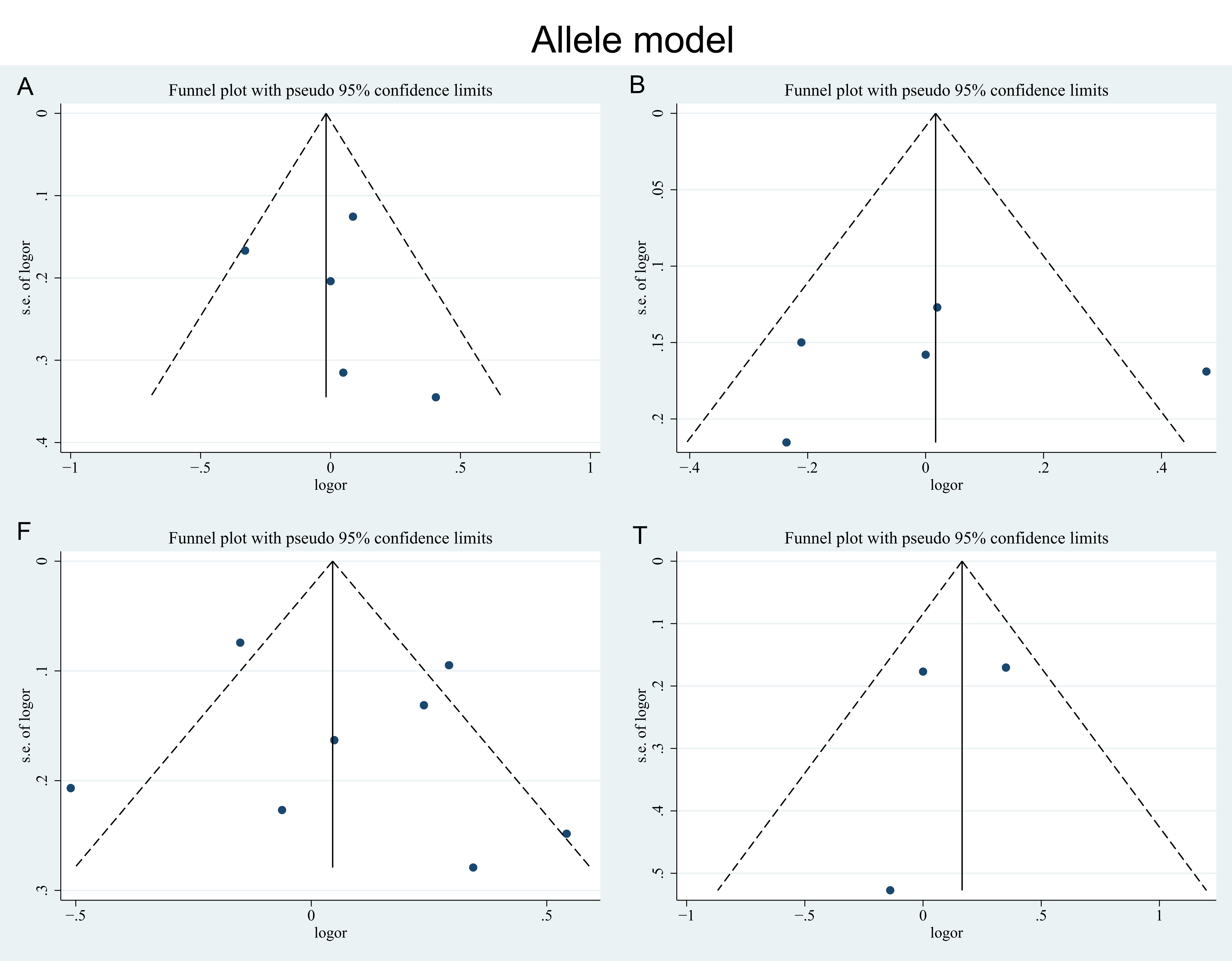

Supplement: Supplemental Information 5 — (A) the ApaI polymorphism; (B) the BsmI polymorphism; (F) the FokI polymorphism; (T) the TaqI polymorphism. [file peerj-11-15181-s005.jpg]

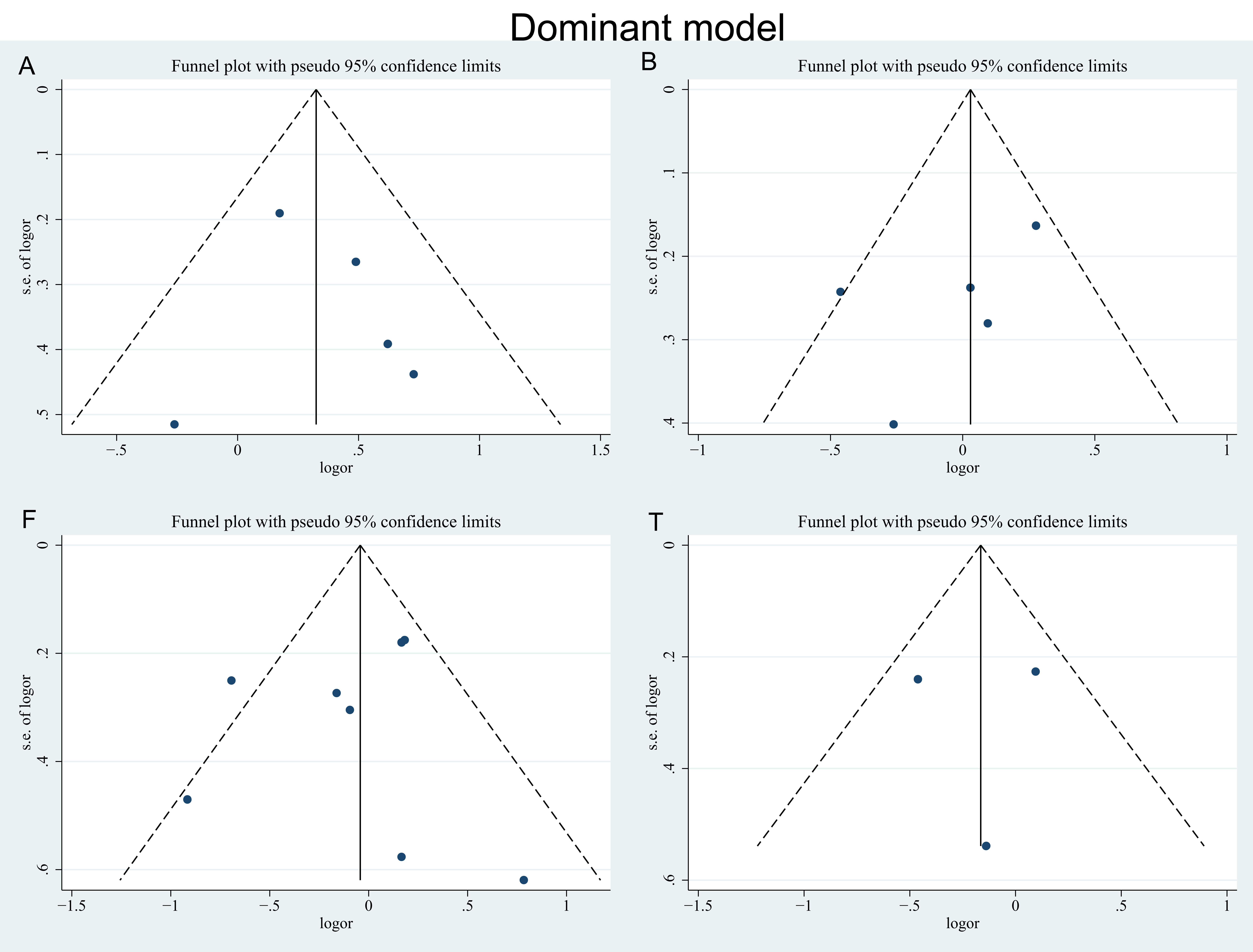

Supplement: Supplemental Information 6 — (A) the ApaI polymorphism; (B) the BsmI polymorphism; (F) the FokI polymorphism; (T) the TaqI polymorphism. [file peerj-11-15181-s006.jpg]

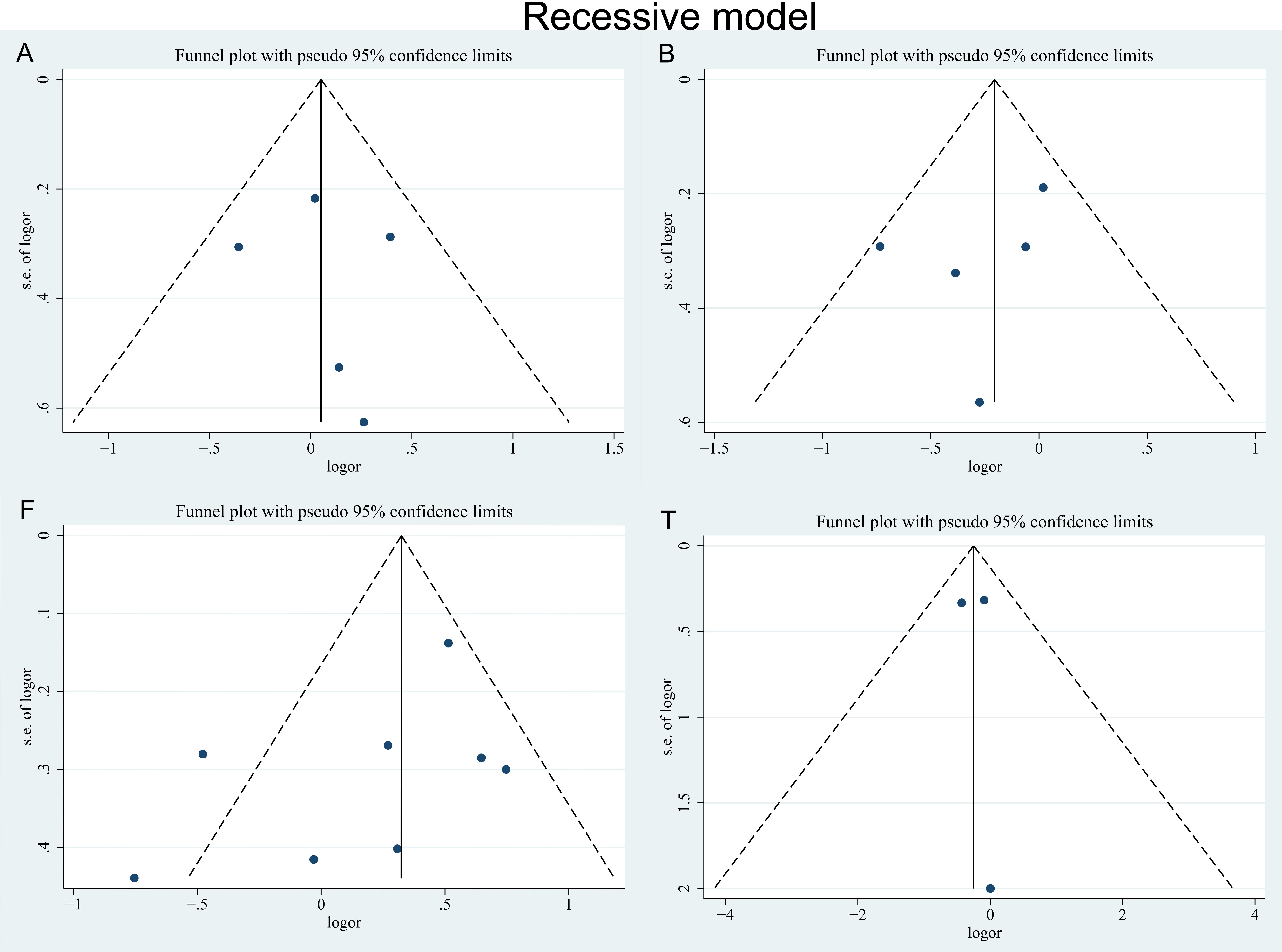

Supplement: Supplemental Information 7 — (A) the ApaI polymorphism; (B) the BsmI polymorphism; (F) the FokI polymorphism; (T) the TaqI polymorphism. [file peerj-11-15181-s007.jpg]

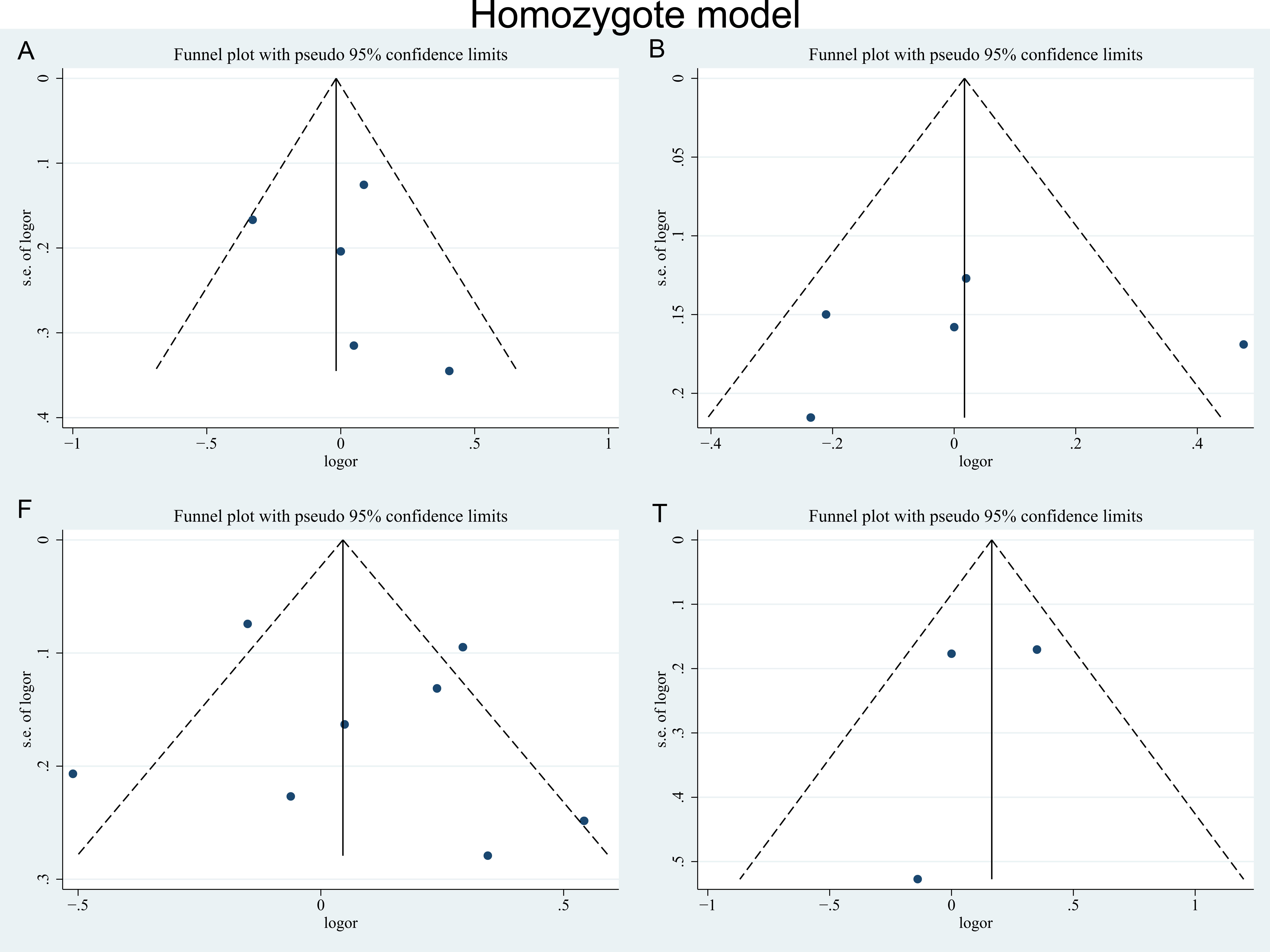

Supplement: Supplemental Information 8 — (A) the ApaI polymorphism; (B) the BsmI polymorphism; (F) the FokI polymorphism; (T) the TaqI polymorphism. [file peerj-11-15181-s008.jpg]

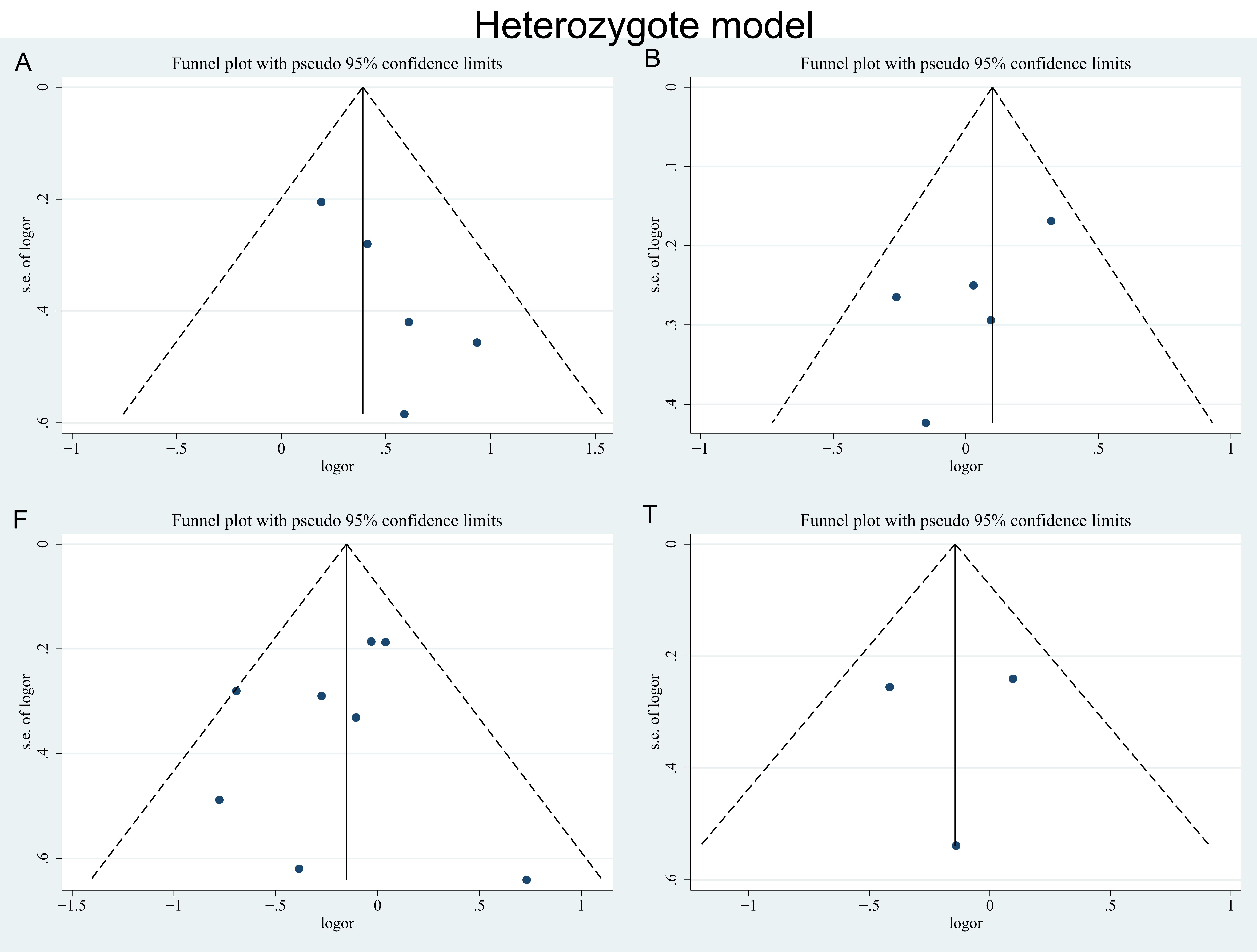

Supplement: Supplemental Information 9 — (A) the ApaI polymorphism; (B) the BsmI polymorphism; (F) the FokI polymorphism; (T) the TaqI polymorphism. [file peerj-11-15181-s009.jpg]
