# Supplementary material for: Association between Vitamin D receptor (VDR) gene polymorphisms and hypertensive disorders of pregnancy: a systematic review and meta-analysis
Source: PeerJ. 2023 Apr 25;11:e15181. doi: 10.7717/peerj.15181 (PMC10143592; doi:10.7717/peerj.15181)
Supplement: Supplemental Information 10 — The contribution this makes to knowledge in light of previously published related reports, including other meta-analyses and systematic reviews. [file peerj-11-15181-s010.docx]

**Association between *Vitamin D receptor (VDR)* gene polymorphisms and** **hypertensive disorders of pregnancy****:** **A** **systematic review and meta-analysis**

**Systematic Review and Meta-Analysis Rationale**

1. The rationale for conducting the meta-analysis

Vitamin D status has been considered an important, modifiable nutrition-related risk factor for hypertensive disorders of pregnancy (HDP) in recent studies (Bodnar et al. 2014; Tabesh et al. 2013). Epidemiologic investigations indicated that vitamin D deficiency or blocked utilization was associated with the increased risk of HDP (Kiely et al. 2016; Serrano et al. 2018), and vitamin D supplementation was confirmed to decrease the risk of preeclampsia when compared to placebo by several meta-analyses (Fogacci et al. 2020; Khaing et al. 2017; Morales-Suárez-Varela et al. 2022; Palacios et al. 2016). The active form of vitamin D, 1,25-Dihydroxyvitamin D_3_ (1,25-(OH)_2_D_3_) mediates its effects of physiological by binding to the vitamin D receptor (VDR) specifically (Haussler et al. 2011). Polymorphisms of the *VDR* gene have been shown to alter VDR functions that affect vitamin D activities and metabolic concentrations (Maestro et al. 2016). Although previous meta-analyses have found that the *VDR* gene polymorphisms could increase the susceptibility to essential hypertension (EH) (Nunes et al. 2020; Zhu et al. 2019), and the *VDR* gene polymorphisms were reported to be associated with plasma renin activity (Vaidya et al. 2011), the relationship between the *VDR* gene polymorphisms and the risk of HDP remains controversial in current studies. The results from current studies are inconsistent between populations from different regions or of different ethnicities. To illustrate the potential association between the *VDR* gene polymorphisms and the risk of HDP susceptibility, we conducted this systematic review and meta-analysis.

1. The contribution that it makes to knowledge in light of previously published related reports, including other meta-analyses and systematic reviews

Considering the significant role of genetic factors in the development of HDP, it is meaningful to identify the associations between the VDR gene polymorphisms and HDP. This is the first meta-analysis to comprehensively investigate the associations between the four common single nucleotide polymorphisms (SNPs) of the *VDR* gene and HDP susceptibility by pooling odds ratios (ORs) and the corresponding 95% confidence intervals (CIs). In conclusion, our current meta-analysis provides evidence that the *VDR* gene *ApaI* and *BsmI* polymorphisms may be associated with the susceptibility risk of HDP. The existing evidence is insufficient to conclude that there are ethnic differences in the association of the *VDR* gene polymorphisms with HDP. Our findings further help the illustration of this association.

**Reference**

Bodnar LM, Simhan HN, Catov JM, Roberts JM, Platt RW, Diesel JC, and Klebanoff MA. 2014. Maternal vitamin D status and the risk of mild and severe preeclampsia. *Epidemiology* 25:207-214. 10.1097/ede.0000000000000039

Fogacci S, Fogacci F, Banach M, Michos ED, Hernandez AV, Lip GYH, Blaha MJ, Toth PP, Borghi C, and Cicero AFG. 2020. Vitamin D supplementation and incident preeclampsia: A systematic review and meta-analysis of randomized clinical trials. *Clin Nutr* 39:1742-1752. 10.1016/j.clnu.2019.08.015

Khaing W, Vallibhakara SA, Tantrakul V, Vallibhakara O, Rattanasiri S, McEvoy M, Attia J, and Thakkinstian A. 2017. Calcium and Vitamin D Supplementation for Prevention of Preeclampsia: A Systematic Review and Network Meta-Analysis. *Nutrients* 9. 10.3390/nu9101141

Kiely ME, Zhang JY, Kinsella M, Khashan AS, and Kenny LC. 2016. Vitamin D status is associated with uteroplacental dysfunction indicated by pre-eclampsia and small-for-gestational-age birth in a large prospective pregnancy cohort in Ireland with low vitamin D status. *Am J Clin Nutr* 104:354-361. 10.3945/ajcn.116.130419

Morales-Suárez-Varela M, Uçar N, Soriano JM, Llopis-Morales A, Sanford BS, and Grant WB. 2022. Vitamin D-Related Risk Factors for Maternal Morbidity and Mortality during Pregnancy: Systematic Review and Meta-Analysis. *Nutrients* 14. 10.3390/nu14194124

Palacios C, De-Regil LM, Lombardo LK, and Peña-Rosas JP. 2016. Vitamin D supplementation during pregnancy: Updated meta-analysis on maternal outcomes. *J Steroid Biochem Mol Biol* 164:148-155. 10.1016/j.jsbmb.2016.02.008

Serrano NC, Guío E, Quintero-Lesmes DC, Becerra-Bayona S, Luna-Gonzalez ML, Herrera VM, and Prada CE. 2018. Vitamin D deficiency and pre-eclampsia in Colombia: PREVitD study. *Pregnancy Hypertens* 14:240-244. 10.1016/j.preghy.2018.03.006

Tabesh M, Salehi-Abargouei A, Tabesh M, and Esmaillzadeh A. 2013. Maternal vitamin D status and risk of pre-eclampsia: a systematic review and meta-analysis. *J Clin Endocrinol Metab* 98:3165-3173. 10.1210/jc.2013-1257
